# Applications of the European Parkinson’s Disease Association sponsored Parkinson’s Disease Composite Scale (PDCS)

**DOI:** 10.1038/s41531-019-0097-1

**Published:** 2019-11-27

**Authors:** Roberta Balestrino, Carlos Alberto Hurtado-Gonzalez, Fabrizio Stocchi, Fabiana Giada Radicati, K. Ray Chaudhuri, Carmen Rodriguez-Blazquez, Pablo Martinez-Martin, The PDCS European Study Group, Astrid D. Adarmes, Carlota Méndez-del-Barrio, Vakirli Ariadne, Zsuzsanna Aschermann, Annamária Juhász, Márk Harmat, Sevasti Bostantjopoulou, Massimo Corbo, Andrea Grassi, Dionysia Dellaporta, Cristian Falup-Pecurariu, Ştefania Diaconu, Nikolaos Giagkou, Alla Guekht, Georgy Popov, Tanya Gurevich, Anders Johansson, Mathias Sundgren, Zinovia Kefalopoulou, John Ellul, Vladimir S. Kostić, Norbert Kovacs, Maria J. Marti, Lluis Planelles, Angel Migirov-Sanderovich, Adi Ezra, Michal Minar, Pablo Mir, Jan Necpal, Maria Popovici, Athima Simitsi, Leonidas Stefanis, Mihaela Simu, Cecilia Rosca, Matej Skorvanek, Alessandro Stefani, Rocco Cerroni, Maria Stamelou, Magda Tsolaki, Vladimira Vuletic, Zoe Katsarou

**Affiliations:** 1 Department of Neuroscience, University of Torino, Turin, Italy; 2 Facultad de Psicología, Universidad Cooperativa de Colombia, Seccional Cali, and Facultad de Medicina, Universidad Libre, Cali, Colombia; 3 Institute for Research and Medical Care, IRCCS, San Raffaele, Rome, Italy; 4 King’s College London, Department of Neurosciences, Institute of Psychiatry, Psychology & Neuroscience, De Crespigny Park, London, SE5 8AF UK; 5 Parkinson Foundation Centre of Excellence, King’s College Hospital, Denmark Hill, London, SE5 9RS UK; 6 National Center of Epidemiology and CIBERNED, Carlos III Institute of Health, Madrid, Spain; 7 Instituto de Biomedicina de Sevilla, Hospital Universitario Virgen del Rocío, CSIC, Universidad de Sevilla, Seville, Spain; 8 Alzheimer Hellas, Thessaloniki, Greece; 9 University of Pecs, Pecs, Hungary; 10 Aristotle University of Thessaloniki, Thessaloniki, Greece; 11 Casa Cura Policlinico, Milan, Italy; 12 Greek Alzheimer Association, Thessaloniki, Makedonia Greece; 13 County Emergency Clinic Hospital Braşov, Transilvania University, Braşov, Romania; 14 Hygeia Hospital, Athens, Greece; 15 Moscow Research and Clinical Center for Neuropsychiatry, Russian National Research Medical University, Moscow, Russia; 16 Neurological Institute, Tel Aviv Medical Center, Sackler School of Medicine & Sagol School of Neuroscience, Tel Aviv University, Tel Aviv, Israel; 17 Karolinska Institutet, Stockholm, Sweden; 18 University Hospital of Patras, Patras, Greece; 19 Institute of Neurology CCS, School of Medicine University of Belgrade, Belgrade, Serbia; 20 University of Pecs, Hungary, and MTA-PTE Clinical MRI Research Group, Pecs, Hungary; 21 Hospital Clinic, IDIBAPS, Barcelona, Catalonia Spain; 22 Movement Disorders Unit, Tel Aviv Medical Center, Tel Aviv, Israel; 23 Comenius University, Bratislava, Slovak Republic; 24 Instituto de Biomedicina de Sevilla, Hospital Universitario Virgen del Rocío, CSIC, Universidad de Sevilla, Seville, and CIBERNED, Spain; 25 Zvolen Hospital, Zvolen, Slovak Republic; 26 Neurotim Med, Timisoara, Romania; 27 Eginition Hospital, National and Kapodistrian University of Athens, Athens, Greece; 28 University of Medicine and Pharmacy Victor Babes, Timisoara, Romania; 29 P. J. Safarik University in Kosice, and University Hospital of L. Pasteur, Kosice, Slovak Republic; 30 Parkinson’s Center, Fondazione Policlinico Tor Vergata, University of Rome Tor Vergata, Roma, Italy; 31 Hygeia Hospital, Athens, Greece and Philipps University, Marburg, Germany; 32 Aristotle University of Thessaloniki, Makedonia, Greece; 33 University Hospital Centre Rijeka, Rijeka, Croatia; 34 Hippokration Hospital, Thessaloniki, Greece

**Keywords:** Parkinson's disease, Epidemiology

## Abstract

This study was addressed to determine the presence of Parkinson disease (PD) manifestations, their distribution according to motor subtypes, and the relationships with health-related quality of life (QoL) using the recently validated European Parkinson’s Disease Association sponsored Parkinson’s Disease Composite Scale (PDCS). Frequency of symptoms was determined by the scores of items (present if >0). Using ROC analysis and Youden method, MDS-UPDRS motor subtypes were projected on the PDCS to achieve a comparable classification based on the PDCS scores. The same method was used to estimate severity levels from other measures in the study. The association between the PDCS and QoL (PDQ-39) was analyzed by correlation and multiple linear regression. The sample consisted of 776 PD patients. We found that the frequency of PD manifestations with PDCS and MDS-UPDRS were overlapping, the average difference between scales being 5.5% only. Using the MDS-UPDRS subtyping, 215 patients (27.7%) were assigned as Tremor Dominant (TD), 60 (7.7%) Indeterminate, and 501 (64.6%) Postural Instability and Gait Difficulty (PIGD) in this cohort. With this classification as criterion, the analogous PDCS-based ratio provided these cut-off values: TD subtype, ≥1.06; Indeterminate, <1.06 but >0.65; and PIGD, <0.65. The agreement between the two scales on this classification was substantial (87.6%; kappa = 0.69). PDCS total score cut-offs for PD severity were: 23/24 for mild/moderate and 41/42 for moderate/severe. Moderate to high correlations (*r* = 0.35–0.80) between PDCS and PDQ-39 were obtained, and the four PDCS domains showed a significant independent influence on QoL. The conclusions are: (1) the PDCS assessed the frequency of PD symptoms analogous to the MDS-UPDRS; (2) motor subtypes and severity levels can be determined with the PDCS; (3) a significant association between PDCS and QoL scores exists.

## Introduction

Parkinson's disease (PD) is the most frequent neurodegenerative movement disorder and the second most frequent neurodegenerative disease worldwide. Although PD is characterized by the loss of dopaminergic neurons in the *substantia nigra*, especially in the *pars compacta*, it is well known that neuroanatomical areas other than the *substantia nigra* and neurotransmitters other than dopamine are involved in its pathogenesis.^1^ PD comprises a range of motor and non-motor characteristics, whose expression vary to some degree among patients.^2,3^ The diagnostic criteria for PD have recently been updated by the Movement Disorders Society (MDS) taking account of this heterogeneity.^4^

Even though PD has historically been defined as a movement disorder, non-motor symptoms (NMS) are an important aspect of the clinical picture.^5,6^ NMS vary from autonomic to gastrointestinal, sleep, sensory, cognitive, and neuropsychiatric disorders. Despite the almost universal presence in PD, their impact on the quality of life (QoL) related to health and disability,^7–9^ misdiagnoses and non-declaration to the health care professionals are frequent^10^ and consequently poorly managed.

At present different scales to assess PD are available: the Movement Disorder Society-Unified Parkinson’s Disease Rating Scale (MDS-UPDRS)^11^ is considered the benchmark for PD assessment, but its use is limited in overloaded clinical settings due to the time taken to complete this instrument; the MDS-UPDRS also includes the Hoehn and Yahr classification to determine the stage of the disease.^12,13^ The Clinical Impression of Severity Index for PD (CISI-PD) evaluates the global severity of PD after the interview and examination;^14^ the Parkinson Disease Questionnaire-39 (PDQ-39) measures the QoL in patients with PD.^15^ The European Parkinson’s Disease Association sponsored Parkinson’s Disease Composite Scale (PDCS) is a recently validated rater-based scale for a rapid evaluation of the most relevant aspects of PD.^16,17^

Given the variety and complexity of PD manifestations, several attempts have been made to identify subtypes of PD patients according to clinical-demographic, pathological, and genetic characteristics. The interest in identifying/defining PD subtypes is based on their possible association with etiological or prognostic aspects and with response to treatment. The most commonly used and accepted classification is based on the UPDRS or MDS-UPDRS scales and defines the clinical subtypes "tremor dominant" (TD), "postural instability and gait difficulty” (PIGD) and "Indeterminate".^18,19^ The PDCS has not been used yet to assess PD severity among different PD subtypes.

A modern definition of PD suggests that PD is a syndromic condition rather than a single entity, and encompasses motor symptoms, NMS, complications and overall impact on activities of daily life.^20^ It is therefore interesting to determine in a simple manner the overall severity of the disorder. To this purpose, cut-off points marking the transition from mild to moderate and moderate to severe burden of the disease have been previously determined for some PD scales,^21^ but not yet for the PDCS.

Finally, as PD severely impacts on patients’ QoL, we explored the relationships between PD-associated factors assessed by the PDCS and the QoL, and how they influence this construct.

The present study was specifically aimed at investigating the following hypotheses: (1) the frequency of PD manifestations detected by the PDCS is similar to the corresponding manifestations detected by the MDS-UPDRS; (2) the scores of the PDCS show a gradient from TD to PIGD subtypes, with Indeterminate cases scoring in between; (3) PD subtypes can be proposed on the bases of PDCS scores; (4) cut-off points can be established for interpreting the PD severity on the basis of the PDCS scores; and (5) a moderate to high association exists between PDCS scores and patients’ QoL.

According to these hypotheses, the following objectives were outlined: (1) to determine the frequency of the PD manifestations detected by the PDCS and compare the results with those of the MDS-UPDRS; (2) to explore the distribution of the PDCS scores according the subtypes of PD (TD, PIGD and indeterminate); (3) to determine cut-off points to identify PD subtypes with the PDCS; (4) to estimate the cut-off values of the PDCS scores for the severity levels mild, moderate, and severe; and (5) to analyze the association between PDCS scores and QoL outcome.

## Results

### Description of the sample

Seven hundred and seventy-six patients from the extensive validation study,^17^ 59% males, were included in the study. 13.7% patients were in HY stage 1; 39.5% in stage 2; 24.5% in stage 3; 16.1% in stage 4; and 6.2% in stage 5. Descriptive characteristics of the sample are shown in Supplementary Table 1 (Supplementary information file).

### Frequency of PD manifestations versus the MDS-UPDRS

The frequency of PD manifestations identified by the PDCS overlapped considerably with related data from the corresponding items of the MDS-UPDRS (Table 1). The discrepancies observed were related with non-equivalent items. For example, the PDCS item 17 “disability” had to be compared with the MDS-UPDRS Part II, which includes 13 items. The average difference in detecting frequency of symptoms or signs between both scales was 5.5%.

**Table 1:** Frequency of manifestations detected by the PDCS compared to the MDS-UPDRS.

Patients with the symptom/sign				Difference (%)
PDCS		MDS-UPDRS		
Item	%	Item	%	
1. Bradykinesia	97.2	Generated	99.7	2.5
2. Tremor	61.8	Generated	67.5	5.7
3. Gait	74.7	3.10	78.5	3.8
4. Balance	61.8	3.12	56.6	5.2
5. Freezing	34.4	3.11	33.0	1.4
6. Nocturnal akinesia	53.2	2.9	68.8	15.6
7. Fatigue	81.4	1.13	76.2	5.2
8. Urinary	64.1	1.10	64.6	0.5
9. Cognition	54.7	1.1	53.9	0.8
10. Depression/Anxiety	70.4	Generated	78.0	7.6
11. Orth. Hypotension	36.8	1.12	49.6	12.8
12. Hallucinations	23.6	1.2	20.6	3.0
13. Dyskinesia	33.0	Generated	33.4	0.4
14. Dystonia	29.1	4.6	29.5	0.4
15. On/Off	48.1	Generated	60.6	12.5
16. Dopamine Dysreg. Synd.	11.6	1.6	10.9	0.7
17. Disability	83.6	Part II	98.3	14.7

*PDCS* Parkinson’s Disease Composite Scale, *MDS-UPDRS* Movement Disorder Society sponsored Unified PD Rating Scale, *Orth. Hypotension* Orthostatic hypotension, *Dopamine Dysreg. Synd.* Dopamine dysregulation syndrome, *Generated* Variable generated in office to be comparable to the corresponding component of the PDCS (see Methods- Data analysis)

### PDCS scores and motor subtypes

According to the MDS-UPDRS, 215 patients (27.7%) were classified as TD, 60 (7.7%) Indeterminate, and 501 (64.6%) PIGD subtype. When the PDCS domains and total scores were broken down by PD subtype, a statistically significant increase in scores (Kruskal–Wallis test, *p* = 0.0001) was observed from TD subtype to PIGD (Table 2), with the intermediate subtype showing intermediate values. The correlation between the respective ratios of the means from “tremor-related/PIGD-related” items from the MDS-UPDRS and PDCS was 0.81 (Spearman coefficient, *p* < 0.0001). On this basis, and taking as criterion the MDS-UPDRS classification, the following ranges and cut-off points were established on the PDCS-based ratio: TD subtype, ≥1.06; Indeterminate, <1.06 but >0.65; and PIGD, <0.65. When the PDCS ratio was broken down according to these cut-off points, 231 patients (29.8%) were TD, 93 (12.0%) Indeterminate, and 452 (58.2%) PIGD. The difference with the MDS-UPDRS-derived subtypes was significant (chi-square, *p* < 0.001); the concordance (Kendall’s coefficient), 0.63 (*p* < 0.0001); the correlation coefficient, 0.70 (*p* < 0.0001); the agreement, 87.6%; and the kappa value was 0.69 (CI95%: 0.66–0.72).

**Table 2:** Parkinson’s Disease Composite Scale (PDCS) scores by subtype of Parkinson’s disease.

Parkinson’s disease subtypes^a^				
PDCS	Tremor predominant	Intermediate	Postural instability and gait difficulty	P*
Motor	6.35 (4.15)	6.78 (4.68)	14.03 (7.22)	0.0001
Non-motor	5.29 (4.52)	5.93 (5.05)	11.04 (7.07)	0.0001
Complications	1.43 (2.42)	2.02 (2.89)	5.06 (4.34)	0.0001
Disability	0.81 (0.90)	1.10 (0.80)	2.47 (1.80)	0.0001
PDCS TOTAL	13.92 (8.86)	15.97 (10.36)	32.28 (16.33)	0.0001

Mean (standard deviation) of scores

*Kruskal–Wallis test

^a^According to the MDS-UPDRS

### PDCS scores and severity levels

Using the severity levels derived from HY, CISI-PD, and MDS-UPDRS,^21^ cut-off points of the PDCS scores were determined by means of ROC analyses (Table 3). Concerning the PDCS total score, the cut-off point between mild and moderate severity levels was established at 23/24 and between moderate and severe at 41/42, as average. Cut-off points were also determined for the PDCS subscales using the severity levels previously established for the MDS-UPDRS corresponding scales^21,22^ (Table 3). The area under the curve of the respective ROC analyses resulted satisfactory as a whole (Supplementary Table 2, Supplementary information file). When the PDCS scores were broken down according the severity levels from other scales (HY, CISI-PD, and MDS-UPDRS), the scores significantly increased with increasing severity level (Table 4). To be highlighted, the maximum difference among the six mean values of the PDCS total score was 4.3 points for mild (17.74–13.48), 6.4 points for moderate (36.46–30.06), and 6.0 points for the severe level (53.46–47.50) (Table 4), representing <7.0% of the maximum total score (observed, 84 points; theoretical, 93).

**Table 3:** Score ranges (cut-off points) for severity levels in the Parkinson’s Disease Composite Scale (PDCS) scores.

Severity levels according to	PDCS Total Score		
	Mild	Moderate	Severe
Hoehn and Yahr Scale	1–21	22–38	≥39
CISI-PD	1–20	21–41	≥42
MDS-UPDRS Part I	1–24	25–47	≥48
MDS-UPDRS Part II	1–25	26–41	≥42
MDS-UPDRS Part III	1–19	20–38	≥39
MDS-UPDRS Part IV	1–26	27–41	≥42
Average	1–23	24–41	≥42
	PDCS Non-Motor subscale		
MDS-UPDRS Part I	1–7	8–14	≥15
	PDCS Disability subscale		
MDS-UPDRS Part II	1	2	≥4
	PDCS Motor subscale		
MDS-UPDRS Part III	1–8	9–18	≥19
	PDCS Complications subscale		
MDS-UPDRS Part IV	1–5	6–8	≥9

Obtained through ROC analysis and Youden method

*CISI-PD* Clinical Impression of Severity Index for Parkinson’s Disease, *MDS-UPDRS* Movement Disorder Society sponsored Unified Parkinson’s Disease Rating Scale

**Table 4:** Parkinson’s Disease Composite Scale (PDCS) scores distribution according to severity levels derived from other scales.

Severity levels from		PDCS score considered	PDCS scores^a^			
			Mild	Moderate	Severe	*p*^b^
Hoehn and Yahr Scale		Total	15.32 ± 9.39	30.06 ± 9.78	47.50 ± 13.50	0.0001
Clinical Impression of Severity Index for Parkinson’s Disease		Total	13.49 ± 7.61	31.99 ± 9.78	53.47 ± 11.88	0.0001
MDS-Unified Parkinson’s Disease Rating Scale	Part I	Total	15.87 ± 10.22	30.42 ± 12.52	51.49 ± 14.69	0.0001
		Non-motor	4.48 ± 3.95	11.09 ± 4.72	20.17 ± 5.02	0.0001
	Part II	Total	14.71 ± 9.10	31.20 ± 12.11	52.57 ± 12.50	0.0001
		Disability	0.87 ± 0.81	2.19 ± 1.39	4.75 ± 1.39	0.0001
	Part III	Total	15.69 ± 9.85	30.18 ± 13.10	50.84 ± 12.51	0.0001
		Motor	6.42 ± 4.44	13.65 ± 5.16	22.44 ± 4.35	0.0001
	Part IV	Total	17.75 ± 11.04	36.46 ± 14.28	51.00 ± 14.05	0.0001
		Complic.	1.37 ± 2.10	6.95 ± 3.08	11.13 ± 3.17	0.0001

*Complic*. Complications

^a^Mean and y standard deviation

^b^Kruskal–Wallis test

### Relationships between PDCS and quality of life

A strong correlation was observed between PDCS (domains and total score) with PDQ-39 Mobility, Activities of daily living, and Summary index (Table 5). Other PDQ-39 domains showed moderate to high correlations with the PDCS as a whole, except “Stigma and Social support”, which reached weak to moderate association. In the multiple regression model, the four PDCS scales showed independent significant influence on the QoL score, prevailing in effect the Non-Motor subscale followed by the Motor one (Table 6).

**Table 5:** Correlations between the Parkinson’s Disease Composite Scale (PDCS) and PDQ-39.

	PDCS				
PDQ-39	Motor	Non- Motor	Complications	Disability	TOTAL SCORE
Mobility	0.75	0.61	0.52	0.75	0.80
ADL	0.67	0.56	0.50	0.70	0.72
Emotional well-being	0.41	0.54	0.29	0.40	0.51
Stigma	0.31	0.30	0.23	0.30	0.35
Social support	0.34	0.37	0.26	0.35	0.39
Cognition	0.46	0.66	0.27	0.50	0.58
Communication	0.52	0.55	0.38	0.53	0.60
Bodily discomfort	0.45	0.51	0.39	0.42	0.54
Summary Index	0.66	0.68	0.48	0.66	0.76

PDQ-39: Parkinson’s Disease Questionnaire-39 items

All coefficients, *p* < 0.0001

**Table 6:** PDCS subscales as determinants of quality of life (PDQ-39 Summary index).

	Coefficient	S.E.	95% CI	*t*	*p*	Beta
Motor	0.65	0.10	0.44-0.85	6.18	<0.001	0.25
Non-Motor	1.04	0.09	0.87-1.21	12.10	<0.001	0.38
Complications	0.36	0.13	0.10-0.62	2.73	0.007	0.08
Disability	2.35	0.45	1.46-3.23	5.21	<0.001	0.21

S.E.: standard error. 95% CI: 95% confidence interval

F (4, 661): 263.08; *p* < 0.0001; Adjusted R-squared: 0.61

Variance inflation factor: 1.50–2.89 (mean: 2.21)

## Discussion

The aim of this study was to provide additional information on the PDCS, a new clinical scale for a global evaluation of PD severity which has been recently validated in two international studies.^16,17^

In the first instance, the frequency of the PD symptoms detected by the PDCS and the MDS-UPDRS was compared: it was almost identical, with the exception of the aspects that are evaluated by the MDS-UPDRS but not by the PDCS, showing a mean discrepancy of only 5.5%. It needs to be recognized however that some non-conformities arise from the intrinsic structural differences between the two instruments, especially their extension format: in fact, the PDCS is a shorter scale and has been specifically designed in order to perform a rapid and real life comprehensive motor and non-motor assessment of the patient and the focus of PDCS is to evaluate broad constructs with a relatively low number of items. Despite this difference in number of items, however, the PDCS showed to be able to correctly assess the aspects that are evaluated by both scales. Therefore, the first hypothesis of this study is confirmed.

Given the growing interest in motor and non-motor subtyping of PD patients,^23,24^ we searched for differences in the PDCS domains and total score among the most commonly used PD subtypes. Patients were divided in motor subtypes (tremor predominant, PIGD, and indeterminate) according to a previously published method.^19^ The PIGD subtype scored significantly higher than the TD in all domains and in the total score of the PDCS; the intermediate group showed intermediate scores between the two groups in all domains and in the total score. These results confirm the second hypotheses of the study and are in agreement with previous evidence on this topic. Several Authors have reported that motor subtypes have different characteristics: PIGD subtype is associated greater disability than other types,^25^ more severe motor complications^26^ and different studies demonstrated that non-tremor dominant subtypes are associated with a broader array and heavier burden of NMS (for a review, see Marras and Chaudhuri^24^). Our data, collected using the PDCS, confirm that PIGD patients have a major burden of motor and NMS, more severe disability and more severe complications. Moreover, the PDCS demonstrated to be able to capture differences between different subtypes of PD in all aspects. In addition, the method for classifying PD patients according to their motor subtype, using the corresponding MDS-UPDRS method as benchmark, was applied. In line with the expectations (third hypothesis of the study), there was a moderate-to-substantial agreement between both scales, a higher concordance being impeded by their structural differences.

The PDCS has been developed to address the unmet need of a rapid and global evaluation tool to give a pragmatic and quick yet reliable measure of motor and NMS in PD patients. The instrument also permits to define global grading scores for PD, which allow capture of disease severity in its complexity and may facilitate a pragmatic, real life management pathway for holistic management of PD in clinical practice. Using pre-existent grading systems,^21^ we defined severity cut-offs in each domain and in the total score of the PDCS. These data allow the clinicians to contextualize the results of the PDCS with additional information on the situation and prognosis of patients, relevant for therapeutic choices. Grading scores are also important for audit of clinical interventions and allow comparison between groups. As such, we feel that the fourth hypothesis of the study was corroborated.

Finally, we analyzed the association between PDCS scores and QoL, which can be defined as the “perception and evaluation by patients themselves of the impact that the disease and its consequences have on their life”.^27^ QoL is a subjective, global and comprehensive patient-reported outcome for both research and management of chronic conditions, such as PD.^28^ The concept of “determinant of QoL” refers to factors keeping a constant and strong association, and a causal relationship with QoL. They are particularly relevant as their modification entails a modification of the QoL, and therefore represent an important therapeutic target and an outcome to assess frequently. We were interested in finding whether the PDCS is capable of measuring aspects that are associated with or can influence the QoL of PD patients. The PDCS showed moderate-high correlations with the PDQ-39, a PD-specific measure of QoL, confirming the fifth hypothesis of the study. All PDCS subscales performed as QoL determinants, in particular the non-motor scale, consistently with previous research. Recent evidence suggests that NMS significantly and independently contribute to worse QoL, and can even exceed the effects of the more traditionally considered motor symptoms;^29,30^ this is especially true for those NMS -such as neuropsychiatric disturbances, cognitive impairment, and fatigue- which have been shown to dramatically impact QoL also in other diseases.^29,31^

Because the assessments were provided in English, a relevant limitation was that patient-reported outcomes in the study were scored through interview to non-English speaking patients. Nonetheless, it is foreseeable that this limitation could affect mainly to the most sensitive and personal aspects of the evaluation, mainly QoL.^32^ Although different ways of administration can induce a usually systematic error,^33^ some studies have used QoL application by interview^34,35^ and have demonstrated non-significant differences between scores obtained through self-evaluation and interview.^34,36^

In conclusion: (1) the PDCS assessed the frequency of PD manifestations similarly to the MDS-UPDRS; (2) motor subtypes and severity levels can be determined with the PDCS, and cut-off values to this purpose are provided; (3) a significant association between PDCS and QoL scores exists. Obviously, future studies to confirm or modify these conclusions are needed.

## Methods

### Design

International, observational, cross-sectional study.

### Patients

The present research was carried out with data from the PDCS Extensive Validation study.^17^ Consecutive patients (*n* = 776) with, a diagnosis of PD, from 11 European countries (Croatia, Greece, Hungary, Israel, Italy, Romania, Russia, Serbia, Slovak Republic, Spain, and Sweden), were included if they met the following inclusion criteria: PD diagnosis by a neurologist, according to internationally recognized criteria;^37^ age ≥ 30 years; and they had granted informed consent to participate. The exclusion criterion was severe concomitant condition hampering proper PD assessment, such as diagnosed severe cognitive impairment, blindness, etc.

### Assessments

Apart from the sociodemographic data and the clinical history of patients with PD, the following evaluations were used:

The PDCS, a simple PD-specific, rater-based scale that assesses the severity of the disease through 17 items grouped into four domains: motor symptoms (6 items), NMSs (6 items), treatment complications (4 items), and disability level (1 item). Item scores are not homogeneous, with items scoring 0–4 and others with different scores (e.g., from 0 up to 7). All items, however, have similar anchors: absent, mild, moderate, severe, and very severe. The total score of the scale ranges from 0 to 93, higher scores indicating higher severity.^16^

The MDS-UPDRS is a scale that evaluates the severity of PD in four domains: Part I—non-motor aspects of daily life experiences (13 items, 0–52 points); Part II—motor aspects of daily life experiences (13 items, 0–52 points); Part III- Motor test (18 items, 0–132 points); and Part IV-Motor complications (6 items, 0–24 points). The higher the score, the greater the severity of the corresponding construct.^11^ The HY scale is used to classify patients in different stages of the disease (from 0, asymptomatic, to 5, wheelchair bound or bedridden unless aided) and the current version is included in the MDS-UPDRS.^12,13^ The CISI-PD evaluates the global severity of PD after the interview and examination;^14^ it consists of four item-domains (motor examination, disability, motor complications, and cognitive impairment). The score of each item ranges from 0 (normal) to 6 (severe), the total score of the scale oscillating between 0 and 24 points.

The PDQ-39 is a specific scale to evaluate the QoL in patients with PD.^15^ It consists of 39 items, grouped into 8 domains: mobility (10 items), AVD (6 items), emotional well-being (6 items), stigma (4 items), social support (3 items), cognition (4 items), communication (3 items) and body discomfort (3 items). The scores of each item run from 0 (normal) to 4 (always). The score of each domain is calculated as a percentage of the maximum possible score and the PDQ-39 Summary Index (PDQ-39 SI) is obtained as the arithmetic mean of the domain scores.

### Data analysis

Local anonymized datasets were sent out to the National Center of Epidemiology, Carlos III Institute of Health (Madrid, Spain) to build the study’s database. Descriptive statistics (i.e. measures of central tendency and dispersion, proportions) were used to characterize the sample. The Shapiro-Francia test showed that the data were not normally distributed.

1. The frequency of each aspect of the disease assessed by the PDCS and the MDS-UPDRS was expressed by the percentage of patients with a score ≥1 (indicating presence of the manifestation). Given that in the PDCS there are items encompassing several items of the MDS-UPDRS, combined scores (sum) of the latter were calculated to compare with the corresponding scores of the PDCS. These combinations were: PDCS Motor examination-Bradykinesia: MDS-UPDRS Part III-Items 4, 5, 6, 7, 8, and 14; PDCS Motor examination-Tremor: MDS-UPDRS Part III-Items 15, 16, 17, and 18; PDCS Non-Motor symptoms-Depression/Anxiety: MDS-UPDRS Part I-Items 3 and 4; PDCS Treatment complications-Dyskinesias: MDS-UPDRS Part IV-Items 1 and 2; and PDCS Treatment complications-ON/OFF: MDS-UPDRS Part IV-Items 3 and 4.
2. The sample was categorized into subtypes (TD, PIGD, and Indeterminate) on the MDS-UPDRS scores, following the method proposed by Stebbins et al.^19^ Kruskal-Wallis equality-of-populations rank test was used to assess differences in the PDCS domains and total score among the three subtypes. In addition, a ratio between means of the PDCS items Tremor/(Gait + Balance/Postural instability + Freezing), similar to the ratio of the MDS-UPDRS used to determine the PD subtypes, was calculated. Next, this ratio was broken down according to the established MDS-UPDRS subtypes, by means of ROC analysis, to determine cut-off points and, subsequently, the subtypes based on the PDCS scores. The concordance and correlation between both subtype classifications (MDS-UPDRS- and PDCS-based) was examined by means of weighted kappa with quadratic weights, Kendall’s concordance and Spearman rank correlation coefficients. Considering the structural differences between the scales, it was hypothesized a moderate to high agreement of both classifications.
3. Severity categories (mild, moderate, and severe) were established in the sample according to the other scales in the study that had previously defined cut-off points for this classification (HY, CISI-PD, MDS-UPSDRS).^21,22^ To determine cut-off points in the PDCS and subscales total scores to group the patients according to these severity levels, ROC analyses of the PDCS scores were applied in each of these scenarios. The cut-off points of the PDCS scores were identified as those with simultaneous maximum sensitivity and specificity between mild and moderate and between moderate and severe. Later, a comparison between the PDCS scores corresponding to the three severity levels established by the other scales was carried out (Kruskal-Wallis test).
4. The association between the PDCS (total score and domains) and the QoL (PDQ-39 components) was explored by means of the Spearman rank correlation coefficient. Coefficient values >0.50 were deemed strong association and 0.35-0.50, moderate correlation.^38^ To establish the role of the PDCS subscales as determinants of the QoL, a multiple linear regression model was built with PDQ-39 SI as dependent variable and the PDCS subscales as independent variables. Multicollinearity among the independent variables was explored by correlation coefficients (*r* < 0.80) and postestimation variance inflation factor.

Data analysis was performed with Stata 15.1 (Stata Corp., College Station, Texas 77845 USA).

### Ethical aspects

The study was approved by the Ethics Committee of all participating sites. Patients were included into the study after providing their signed informed consent.

### Reporting summary

Further information on experimental design is available in the Nature Research Reporting Summary linked to this article.

## Supplementary information

Supplemental Material

Reporting Summary

## Data Availability

The datasets generated and/or analyzed during the current study are available from the corresponding author on reasonable request.
