# Supplementary material for: Applications of the European Parkinson’s Disease Association sponsored Parkinson’s Disease Composite Scale (PDCS)
Source: NPJ Parkinsons Dis. 2019 Nov 27;5:26. doi: 10.1038/s41531-019-0097-1 (PMC6881347; doi:10.1038/s41531-019-0097-1)
Supplement: Supplementary file 1 — Supplemental Material [file 41531_2019_97_MOESM1_ESM.pdf]

Supplementary Information

Supplementary Table 1 - Descriptive statistics of the sample

**Category**	**Mean ± SD**	**Range**
Age	67.94±9.96	39-95
Age at Onset	59.05±11.04	20-89
Parkinson's disease duration	8.90±6.30	0-49
Levodopa-equivalent daily dose	732.67±581.18	0-4066
**MDS- Unified Parkinson's Disease** Rating Scale Part I	12.10±7.69	0-41
MDS- Unified Parkinson's Disease Rating Scale Part II	15.35±11.04	0-48
MDS- Unified Parkinson's Disease Rating Scale Part III	35.60±19.35	2-109
MDS- Unified Parkinson's Disease Rating Scale Part IV	4.39±4.83	0-20
**Parkinson's Disease Composite Scale** - Total Score	26.16±16.87	1-84
Motor	11.37±7.30	0-31
Non-Motor	9.04±6.84	0-32
Complications	3.85±4.21	0-19
Disability	1.90±1.72	0-6
**PD Questionnaire-39 items (PDQ-39)** Summary Index	27.63±18.50	0-100
Mobility	37.20±30.97	0-100
Activities of Daily Living	34.99±28.62	0-100
Emotional Well-being	30.26±22.85	0-100
Stigma	24.84±25.21	0-100
Social Support	14.84±19.55	0-100
Cognition	26.65±22.54	0-100
Communication	21.28±21.88	0-100
Discomfort	30.64±23.97	0-100
**Clinical Impression of Severity Index for PD -** Total Score	8.64±5.16	0-24
Motor Signs	3.05±1.39	0-6
Disability	2.73±1.51	0-6
Motor Complications	1.55±1.81	0-6
Cognitive Status	1.31±1.49	0-6

SD: Standard deviation. PD: Parkinson's disease. MDS: Movement Disorder Society.

Supplementary Table 2 – Area under the curve from ROC analysis by severity level

Severity levels according to	PDCS Total Score	
	**Mild - Moderate**	**Moderate - Severe**
Hoehn and Yahr Scale	0.87	0.86
CISI-PD	0.93	0.91
MDS-UPDRS Part 1	0.82	0.85
MDS-UPDRS Part 2	0.82	0.89
MDS-UPDRS Part 3	0.81	0.87
MDS-UPDRS Part 4	0.79	0.77
	**PDCS Non-Motor subscale**	
MDS-UPDRS Part 1	0.85	0.90
	**PDCS Disability subscale**	
MDS-UPDRS Part 2	0.73	0.87
	**PDCS Motor subscale**	
MDS-UPDRS Part 3	0.85	0.90
	**PDCS Complications subscale**	
MDS-UPDRS Part 4	0.86	0.85
